# Fatty acid composition, antioxidant and antibacterial activities of *Adonis wolgensis* L. extract

**Published:** 2014

**Authors:** Maryam Mohadjerani, Rahmatollah Tavakoli, Rahman Hosseinzadeh

**Affiliations:** 1 *Department of Molecular and Cell Biology, Faculty of Basic Sciences, University of Mazandaran, Babolsar,I. R. Iran*; 2 *Department of Organic Chemistry, Faculty of Chemistry, University of Mazandaran, Babolsar, I. R. Iran*; 3 *Department of Medical Engineering, Maziar University, Noor, I. R. Iran*

**Keywords:** *Adonis wolgensis*, *Antibacterial activity*, *Antioxidant activity*, *Fatty acid*

## Abstract

**Objectives:** The objective of this study was to analyze the fatty acid content, antioxidant, and antibacterial activities of hydro-methanolic extract of *Adonis wolgensis* L. (*A. wolgensis* L.) growing wild in north of Iran.

**Materials and Methods:** Oils of *A. wolgensis* L. was obtained by means of Soxhlet apparatus from leaves and stems. Methyl esters were derived from the oily mixtures by *trans*-esterification process and were analyzed by GC/FID and GC/MS systems. Phenolic compounds extraction was done with aqueous methanol (90%). This extract was investigated for antioxidant activity using DPPH radical scavenging and reducing power methods and was also tested against a panel of microorganisms.

**Results:** Linolenic acid (45.83%) and oleic acid (47.54%) were the most abundant fatty acids in leaves and stems, respectively. Hydro-methanolic extract with the high amount of total phenolics (9.20 ±0.011 mg GAE per dry matter) was the potent antioxidant in the assays. Results obtained from measurements of MIC for extract, indicated that *E. coli*, *S. aureus*, and *S. enteritidis* were the most sensitive microorganisms tested, but no activity was observed against Gram-positive microorganism (*B. subtilis*).

**Conclusion:** The results obtained from the present study indicated that the oil of *A. wolgensis* leaves and stems contained a high source of poly-unsaturated fatty acids (PUFAs). These results also showed that hydro-methanolic extract of this plant contained significant antioxidant and antibacterial activities.

## Introduction

Plants are rich sources of beneficial secondary metabolites which are attractive as pharmaceuticals, antimicrobials, flavors, fragrances, and pesticides. Among these herbal constituents, fatty acids, antioxidants, and antibacterial compounds play very important role in maintaining health and improving the quality of human life. Therefore, there is a growing interest in finding plants for food and medicinal applications (Tavakoli et al., 2012a ▶; Jelodarian et al., 2011 ▶; Sedighinia et al., 2012 ▶; Tekeli et al., 2010 ▶).

The genus *Adonis *L. (Ranunculaceae) comprises about 30 species of herbaceous plants, which are mainly distributed in the temperate regions of Asia and Europe (Wang, 1994 ▶). Nine taxa of this genus have already been reported from flora of Iran (Rechinger, 1992 ▶). A literature survey showed that the *Adonis* species has been found to be rich in flavonoids (Komissarenko et al., 1973 ▶), cardenolides (Pauli et al., 1993 ▶), and phenolic glycosides (Pauli and Junior, 1995 ▶). Recently, we reported essential oil composition of *A. wolgensis* L. aerial parts (Tavakoli et al., 2012b ▶). There are no available data in the literature on the analysis of fatty acid’s components, antioxidant, and antibacterial activities of *A. wolgensis* L. The purpose of the present study is to evaluate the chemical composition of fatty acids, antioxidant, and antibacterial activities of hydro-methanolic extract of aerial parts of *A. wolgensis* L. grown in the north of Iran.

## Material and Methods

**Plant materials**

The aerial parts of *Adonis wolgensis* L. were collected during May 2011 from Alborz Mountains (Mazandaran province, Iran). The identification was done by one of the authors and voucher specimens were deposited in the herbarium of the Department of Biology, Faculty of Science, University of Mazandaran, Babolsar (No. 1502). The plant material was air-dried at room temperature and protected from light over a week.

**Chemicals**

Ascorbic acid (AA), 2,2-diphenyl-1-picrylhydrazyl (DPPH), gallic acid, Folin-Ciocalteu's reagent, FeCl_3_, and trichloroacetic acid (TCA) were obtained from Sigma and all other chemicals from Fluka BioChemika or Merck.

**Oil extraction and fatty acid methyl esters preparation**

Dried ground plant materials (leaf and stem) were extracted with hexane using a Soxhlet apparatus (70 °C, 6 h) to obtain the lipid components. After removing hexane using rotary evaporator, the oily mixtures were derived to their methyl esters by *trans*-esterification process (Paquat and Hautfenne, 1992 ▶) and were analyzed by GC/FID and GC/MS systems. 

**Analysis of fatty acid methyl esters**

Gas chromatography analyses were performed with a HP 5890 Series II gas chromatograph equipped with a FID detector and HP-5 (5% phenyl/95% polydimethylsiloxane) fused silica capillary column (30×0.25 mm^2^ i.d., film thickness 0.25 µm) using helium as carrier gas (1.0 mL/min). The injector temperature was 250 ºC and the column oven programmed was 50-220 ºC at 4 °C/min. The detector (FID) was operated at 260 ºC. The GC/MS was performed with an Agilent 5973MSD coupled to an Agilent 6890 gas chromatograph, using helium as carrier gas, and the same column and oven conditions as above. The quadrupole mass spectrometer was scanned over the 35-500 amu with an ionizing voltage of 70 eV and an ionization current of 150 µA. Identification of constituents was done by matching their mass spectra with those in the Wiley 7 and NIST (National Institute of Science and Technology) libraries.

**Preparation of extract**

For preparation of extract, ground material (10 g) was extracted in 90% aqueous methanol (100 ml) using an orbital shaker for 24 h at room temperature. The extract was separated from solid by filtration. The remaining residue was re-extracted twice and the extracts were combined.

**Assessment of total phenolic content**

Total phenolic constituents of the hydro-methanolic extract were determined according to the literature methods involving the Folin-Ciocalteu reagent and gallic acid as standard (Singleton and Rossi, 1965 ▶). Twenty microliters of extract solution (50 μg of dried extract) was taken in a cuvette, then 1.58 ml of distilled water and 100 μl of Folin-Ciocalteu reagent were added, and cuvette was shaken thoroughly. After 3 min, 300 μl of the sodium carbonate solution (7% w/v) was added, and the mixture was allowed to stand for 2 h with intermittent shaking. All of the experiments were conducted in triplicate. Absorbance was measured at 760 nm.

**DPPH radical scavenging assay**

The ability of extract to scavenge DPPH radicals was determined according to the method of Blois (1958) ▶. Briefly, 1 ml of a 1-mM methanolic solution of DPPH was mixed with 3 ml of extract solution in methanol (containing 25-200 μg of dried extract). The mixture was then vortexed vigorously and left for 30 min at room temperature in the dark. The absorbance was measured at 517 nm and activity was expressed as percentage DPPH scavenging relative to control using the following equation:

I (%) = 100 × (*A*_blank_* – A*_sample_*/A*_blank_)

Where *A*_blank_ is the absorbance of the control reaction (containing all reagents except the test compound) and *A*_sample_ is the absorbance of the test compound. Extract concentration providing 50% inhibition (IC_50_) was calculated from a graph plotting percentage inhibition against extract concentration. All of the experiments were conducted in triplicate.

**Determination of reducing power**

Ability of the extract to reduce iron (III) was assessed by the method of Yildirim, Mavi, and Kara (2001) ▶. The dried extract (50-600 μg) in 1 ml of methanol was mixed with 2.5 ml of phosphate buffer (0.2 M, pH 6.6) and 2.5 ml of potassium ferricyanide (K_3_Fe (CN)_6_, 10 g/l), then the mixture was incubated at 50 ºC for 30 min. After incubation, 2.5 ml of trichloroacetic acid (100 g l^-1^) was added. Finally, 2.5 ml of the solution was mixed with 2.5 ml of distilled water and 0.5 ml of FeCl_3 _(1 g l^-1^) and the absorbance was measured at 700 nm. Ascorbic acid was used as standard. All of the experiments were conducted in triplicate. Higher absorbance indicates higher reducing power.

**Antibacterial activity**

The hydro-methanolic extract was tested against a panel of microorganisms, including *Escherichia coli *ATCC 1533*, Bacillus subtilis* ATCC 1156, *Staphylococcus aureus* ATCC 1189, and *Salmonella enteritidis* ATCC 1609. For calculation of minimum inhibitory concentration (MIC) which represents the concentration that completely inhibits the growth of microorganisms, a microdilution broth susceptibility assay was used, as reported by Clinical and Laboratory Standards Institute (CLSI, 1999). 

All tests were performed in Mueller Hinton Broth. A series of dilutions were prepared in the range 0.01-72 mg/ml of the extract in a 96-well microtiter plate, including one growth control (NB + Tween 80) and one sterility control (NB + Tween 80 + extract). One hundred and sixty μl NB was added onto the microplates with 20 μl of the tested solution. Then, 20 μl 5 × 10^5^ CFU/ml (confirmed by viable count) of standard microorganism suspension was inoculated onto the microplates. The plates were incubated at 37 °C for 24 h. Amoxicillin was used as a reference compound for antibacterial activity. The growth was indicated by the presence of a white ‘pellet’ on the well bottom. The MIC was calculated as the highest dilution showing complete inhibition of the tested strains. All of the experiments were conducted in triplicate.

## Results

**Fatty acid composition**

Fatty acid composition of *A. wolgensis* L. is presented in Table 1. As shown in this table, palmitic acid (28.25% in leaf and 33.10% in stem) and linoleic acid (12.55% in leaf and 11.26% in stem) were similar in both parts. However, α-linolenic acid (45.83%), as a major component, was only found in leaf extract and oleic acid (47.54%), as a major component, only was found in stem extract Table 1.

**Table 1:** Fatty acids detected in the leaf and stem extracts of *Adonis wolgensis* L

**Fatty acids**	**RT** a	**Leaf (%)**	**Stem (%)**
**Palmitic acid**	34.48	28.25	33.10
**Linoleic acid**	37.54	12.55	11.26
**Oleic acid**	37.64	-	47.54
**α-Linolenic acid**	37.78	45.83	-
**Other hydrocarbon compounds identified**		13.37	8.1
**∑** **Saturated fatty acid**		28.25	33.10
**∑** **Unsaturated fatty acids**		58.38	58.8

a RT: Retention time

**Antioxidant activity**

Results of the colorimetric analysis of total phenolics, based on the absorbance values of the extract solutions and comparison with the standard solutions of gallic acid equivalents, are given in Table 2. Total phenolic content (TPC) of hydro-methanolic extract of *A. wolgensis* L. was 9.20±0.011 GAE/g dry matter as illustrated in Table 2.

Free radical scavenging capacity of the hydro-methanolic extract is also shown in Table 2. Since the reaction followed a concentration-dependent pattern, only concentration of active extract providing 50% inhibition concentration (IC_50_) was included in the table. Ascorbic acid was used as a standard. The value IC_50_ of *A. wolgensis* L. was 27.45±0.083 (*µ*g/ml).

Results of reducing power analysis of hydro-methanolic extract of *A. wolgensis* L. is presented in Table 3. The reducing potential of this extract measured for the concentration up to 0.6 mg/ml showed general increase in activity when concentration increased. The reducing potential of the tested hydro-methanolic extract was observed at concentrations of 0.05-0.6 mg/ml. The absorbance recorded for the tested extract solutions in this assay was in between 0.18 and 1.96. In comparison with standard ascorbic acid and gallic acid, hydro-methanolic extract showed lower reducing power as illustrated in Table 3.

**Table 2:** Total phenolic content and radical scavenging capacity of hydro-methanolic extract of* Adonis wolgensis* L^a^

**Samples**	**Gallic acid equivalents** b	**IC**_50_ ** (** ***µ*** **g/ml)**
Hydro-methanolic extract	9.20±0.011	27.45±0.083
Ascorbic acid	-	22.23±0.175

a Values are mean±SD of three separate experiments;

b mg GAE per dry matter.

**Table 3:** Reducing potential of Hydro-methanolic extract of *Adonis wolgensis* L a^,^^b^

**Concentration (mg/ml)**	**0.05**	**0.1**	**0.2**	**0.4**	**0.6**
Absorbance	0.18±0.01	0.37±0.011	0.68±0.01	1.32±0.02	1.96±0.12

a Absorbance values at 700 nm;

b Values are mean±SD of three separate experiments.

**Antibacterial activity**

In this study, the antibacterial activity of the hydro-methanolic extract of *A. wolgensis* L. against panel pathogenic microorganisms was assessed by measurement of minimum inhibitory concentration (MIC). The results are presented in Table 4. From the results given in this table, we can conclude that the extract of *A. wolgensis* was particularly effective against the Gram-negative *Salmonella enteritidis *(48±1.56 *µ*g/ml) and *Escherichia coli* (50±1.94 *µ*g/ml), and the Gram-positive *Staphylococcus aureus* (50±1.83 *µ*g/ml), but no activity was observed against Gram-positive microorganism *Bacillus subtilis* (Table 4).

**Table 4:** Antibacterial activity in terms of MIC of hydro-methanolic extract of *A. wolgensis* L. on the selected strains of bacteria a^,^^b^

**Tested microorganisms**	**Extract**	**Amoxycillin**
***Salmonella enteritidis***	48±2.56	25±1.71
***Staphylococcus aureus***	50±2.83	28±1.82
***Escherichia coli***	50±3.94	28±1.75
***Bacillus subtilis***	-	30±2.54

a Minimum inhibitory concentration, MIC (*µ*g/ml).

b Values are mean ±SD of three separate experiments.

## Discussion

According to the results obtained from GC-MS, the leaf and stem of the plant differed in terms of fatty acid content and their percentages (Table 1). Table 1 shows that the unsaturated fatty acid contents were higher than saturated ones in both tested extracts. Poly-unsaturated fatty acids (PUFAs) are considered valuable compounds in the human diet because of their effect in human health (Das, 2000 ▶; Grundy, 1997 ▶). Therefore, increased consumption of monounsaturated fatty acids and polyunsaturated fatty acids and decreased consumption of saturated fatty acids are linked to positive health outcomes. According to these results, the oil of *A. wolgensis* L. may be a good source of poly-unsaturated fatty acids.

Aqueous mixtures of methanol, ethanol, and acetone are commonly used to extract plants (Sun and Ho, 2005 ▶). Thus, in this study, 90% methanol was used as extracting solvent. Phenolic compounds are attracting considerable interest in the field of medicine and food chemistry due to their promising antioxidant potential. Therefore, measurement of total phenolic content of natural products is an essential task. The results show that the hydro-methanolic extract of *A. wolgensis* L. is a rich source of polyphenolic compounds (9.20±0.011 GAE/g dry matter), as confirmed by aforementioned investigations (Komissarenko et al., 1973 ▶; Pauli and Junior, 1995 ▶). The content of total phenolic in the present study was comparable with other researches (Silva et al., 2006 ▶; Sultana et al., 2007 ▶).

Free radicals are involved in lipid peroxidation, which plays important role in the development of various chronic diseases such as cancer, Alzheimer's, and heart diseases (Halliwell and Gutteridge, 1989 ▶). Therefore, the ability to scavenge free radicals is an important antioxidant property. The value IC_50_ shows hydro-methanolic extract which is contained high amount of total phenolics, to be also an active radical scavenger.

The reducing power of a compound (Fe^3+^-Fe^2+^) is used to determine the electron-donating capacity of antioxidants and is one of the main characteristics of phenolic compounds (Dorman et al., 2003 ▶). Ability of the extract to reduce iron (III) to iron (II) was determined and compared with that of ascorbic acid and gallic acid which are known to be strong reducing agents. The results of antioxidant activity of hydro-methanolic extract suggest that the antioxidant activity has high correlation with total phenolic content. Several researchers have reported the relationship between total phenolic content and antioxidant activity in plant extracts (Sultana et al., 2007 ▶; Mustafa et al., 2010 ▶). On the other hand, the results of the present study indicated that phenolic compounds are powerful scavengers of free radicals and reducing agents. However, further investigation is needed to identify individual compounds forming antioxidative system and develop their application as food and pharmaceuticals.

Increase of antibiotic resistance as well as undesirable side effects of synthetic drugs have triggered immense interest in the search for new antimicrobial agents of plant origin (Lewis and Elvin-Lewis, 1995 ▶; Alves et al., 2012 ▶). In this study, the antibacterial activity of the hydro-methanolic extract of *A. wolgensis* L. against panel pathogenic microorganisms was assessed by measuring MIC. The results showed that hydro-methanolic extract contains effective antibacterial activity and are comparable with amoxycillin as standard antibiotic and results of others studies (Quereshi et al., 2010 ▶; Miceli et al., 2009 ▶). High antibacterial activity of this extract could be due to the high content of phenolic compounds (Herald & Davidson, 1983 ▶; Stead, 1993 ▶). 

There are reports that show extracts of some plants and mushrooms have poor antibacterial activity against *B. subtilis*, while possess significant activity against other Gram-positive and Gram-negative bacteria as mentioned in this study (Smania et al., 2007 ▶; Alves et al., 2012 ▶). Therefore, further isolation and purification of this extract are required to determine the active compounds responsible for antibacterial activity. Although our results support the idea that *A. wolgensis* L. extract is a candidate for treatment of infectious diseases, clinical trials are required to confirm its antibacterial effect and general safety.
